# A large mediastinal benign myoepithelioma effacing the entire hemithorax: case report with literature review

**DOI:** 10.1186/s13000-015-0340-y

**Published:** 2015-07-14

**Authors:** Atif Ali Hashmi, Amna Khurshid, Naveen Faridi, Muhammad Muzzammil Edhi, Mehmood Khan

**Affiliations:** Department of Histopathology, Liaquat National Hospital and Medical College, Karachi, Pakistan; Intern, Liaquat National Hospital and Medical College, Karachi, Pakistan; Dhaka Medical College, Dhaka, Bangladesh

**Keywords:** Myoepithelioma, Benign mixed tumor, Mediastinum

## Abstract

**Background:**

Myoepithelial neoplasms, although sometimes encountered in soft tissues are described very rarely in mediastinum and lung. We reported a rare case of such a tumor which was very large in size and not connected to respiratory tree.

**Case presentation:**

A 24 year old male presented with blunt chest pain and respiratory distress. A CT scan was performed which showed large heterogeneously enhancing soft tissue mass occupying the left hemithorax. It measures 18.5 X 15.8 X 7.6. Thoracotomy with excision of the tumor was done. Operative findings include multilobulated and nodular large glistening white tumor located in anterior mediastinum adherent to parietal pleura and effacing the pulmonary parenchyma. However tumor was not connected or seems to originate from trachea or lung. Microscopic sections show neoplastic lesion composed of nests, cords and trabeculae of small to medium sized cells with round nuclei and clear cytoplasm. Background showed myxoid appearance with areas of cartilaginous differentiation. Immunohistochemical expression of CKAE1/AE3, p63, ASMA, S100 and GFAP favored the diagnosis of benign myoepithelioma.

**Conclusion:**

Myoepithelial tumors are rare soft tissue tumors thought to arise from stem cells capable of divergent differentiation and occur anywhere in the body. Histopathologic recognition of these tumors is essential as these tumors may behave in a benign fashion despite large sizes.

## Background

Myoepithelial neoplasms are commonly encountered lesions of salivary glands and are rarely seen in soft tissues [1]. On the other hand myoepitheliomas are extremely rare in mediastinum [2]. A few case reports of benign mixed tumors described so far in mediastinum were thought to arise from ectopic salivary gland tissue along tracheobronchial tree and secondarily involved the mediastinum [3]. The term begin mixed tumor is used when there is ductular differentiation. On the other hand, myoepthelioma by definition don’t show obvious ductal differentiation.

Soft tissue myoepitheliomas are increasingly recognized tumors, formerly designated as parachordomas. They occur in all age groups and peak in 2^nd^ to 4^th^ decade of life. There is no significant gender predilection. They usually present with slowly growing painless mass in deep soft tissues of the extremities with head n neck and trunk being other sites involved in decreasing order of frequency.^9^ Grossly myoepitheliomas form nodular masses ranging in size from 1 to 12 cm. They are usually well circumscribed and glistening, myxoid to gelatinous consistency. Histopathologically, a wide spectrum of morphologic appearances can be seen with predominant reticular growth pattern of epitheloid to spindled cells. The background stroma is collagenous to chondromyxoid. Most myoepitheliomas show mild nuclear atypia with low mitotic activity (<1/10HFFs). Histological features indicating adverse prognosis include obvious cytologic atypia (large cells with coarse chromatin/ prominent nucleoli), areas of necrosis, significant mitotic activity and invasive borders. Tumors with these features are termed as malignant myoepithelioma or myoepithelial carcinoma.^9^ Myoeptheliomas consistently co-express epithelial markers (cytokeratin and EMA) and S100. Expression of other myoepithelial markers including GFAP, p63, ASMA, Calponin are seen in a subset of tumors (15-50 %).^9^ Cytogenetically myoepithelial tumors are characterized by EWSR1 gene rearrangements with variety of different fusion partners including EWSR1-PBX1 fusion [t(1;22)(q23;q12)] and EWSR1-ZNF444 fusion [t(19;22)(q13;q12)]. Antonescu et al. in a large series of 66 myoepitheliomas found EWSR1 rearrangement by FISH in 30 (45 %) cases. RT-PCR studies performed on these 30 cases showed EWSR1-POU5F1 (5 cases), EWSR1-PBX1 (5 cases) and EWSR1-ZNF444 (1 case) fusion and 19 cases lacking an identifiable fusion partner [4]. Ultrastructurally cells of myoepithelioma show incomplete epithelial differentiation with primitive cell junctions, fragmented basal lamina and microvillous projections [5].

We described a case of benign myoepithelioma which is unique in the aspect that it was not arising from respiratory tree and was extremely huge in size(18.5 X 15.8 X 7.6 cm) almost effacing the entire hemithorax.

## Case presentation

**Fig. 1:**
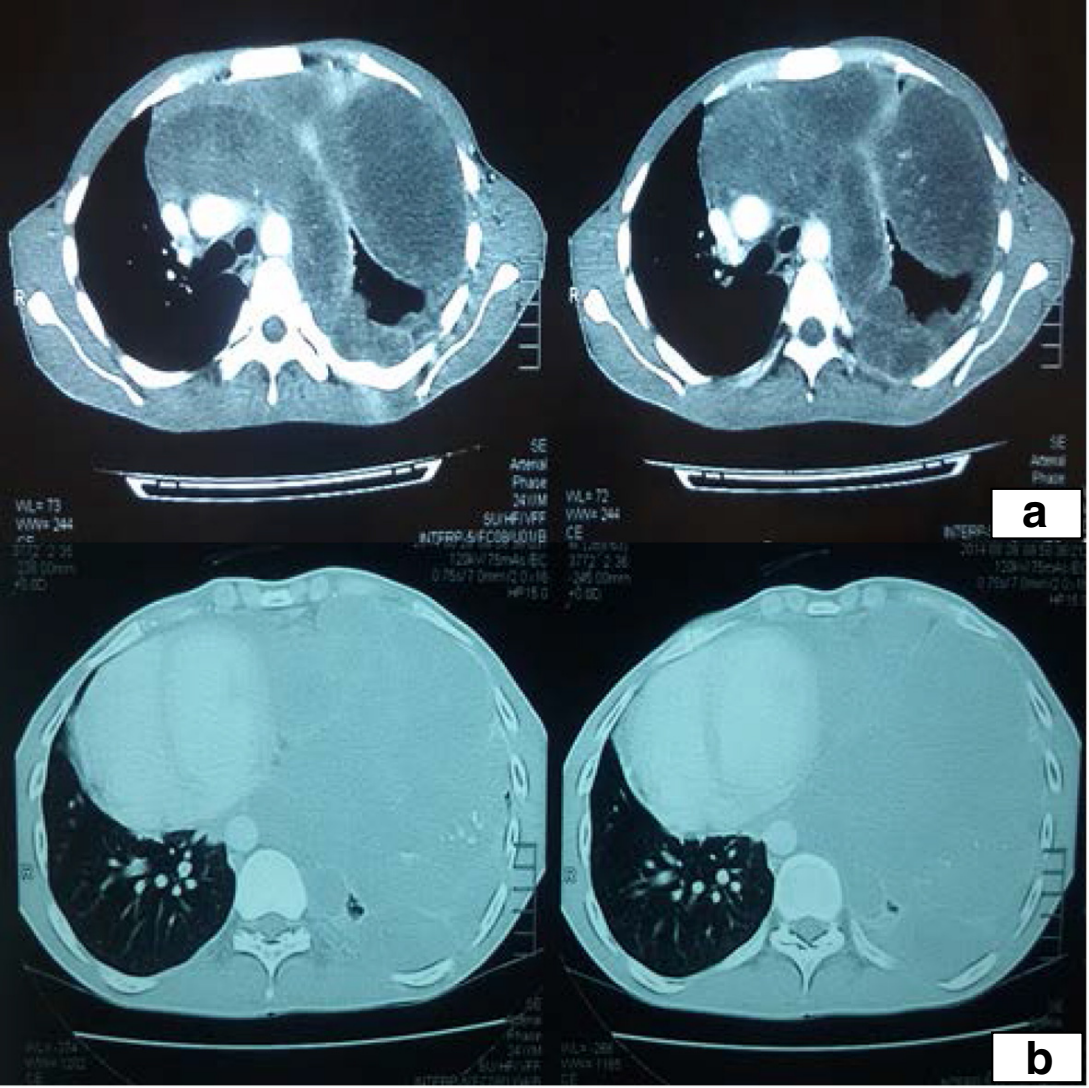
CT scan **a & b**: showing large heterogeneously enhancing mass effacing the left hemithorax and anterior mediastinum

A 24 year old male presented with blunt chest pain and respiratory distress. A CT scan was performed which showed a huge heterogeneously enhancing soft tissue mass having solid component, fluid loculi and amorphous calcifications occupying the left hemithorax. It measures 18.5 X 15.8 X 7.6. The main bulk of the tumor was in the lower half and it reached inferiorly upto the left costophrenic sulcus. It was extending along the pleural surface reaching upto the lung apex. The left lung was almost encased by this mass. Similar huge soft tissue density masses were seen on the mediastinal surface of left sided pleura where it took the form of conglomerate mass in the superior mediastinum and on the lateral aspect of pulmonary trunk and heart. Mediastinum was pushed to right side. The main pulmonary trunk showed extrinsic compression and left main pulmonary trunk was severely compressed. Extrinsic compression of left atrium was also noted. There was no pleural effusion or bony erosion by the mass. Right lung was normal (Fig. 1). In two initial ultrasound guided preoperative needle biopsies, diagnosis of chondroid hamartoma and sarcomatoid mesothelioma were favored from outside institutions. Bronchoscopy didn’t reveal any intraluminal growth. Metastatic workup including CT scan abdomen and bone scan were unremarkable. Serum tumor markers including lactate dehydrogenase, alpha fetoprotein, beta human chorionic gonadotropin were within normal limits. Thoracotomy with excision of the tumor was planned and intraoperative consultation was requested in which an initial diagnosis of chondroid neoplasm was given with final diagnosis deferred till permanent sections.

Operative findings include multilobulated and nodular large glistening white tumor located in anterior mediastinum adherent to parietal pleura and effacing the pulmonary parenchyma. However, tumor didn’t appear to invade the lung parenchyma and there was no trachea-bronchial connection. Borders of the tumor were well defined. Tumor was not firmly adherent to the mediastinal structures, pericardium and pleura and was easily scooped out during surgery. There was no apparent invasion into any of the adjacent structures. During surgery left thoracotomy incision was given and pleural cavity is entered through 7^th^ intercostal space. Excision of the tumor was done and cavity was repaired.

**Fig. 2:**
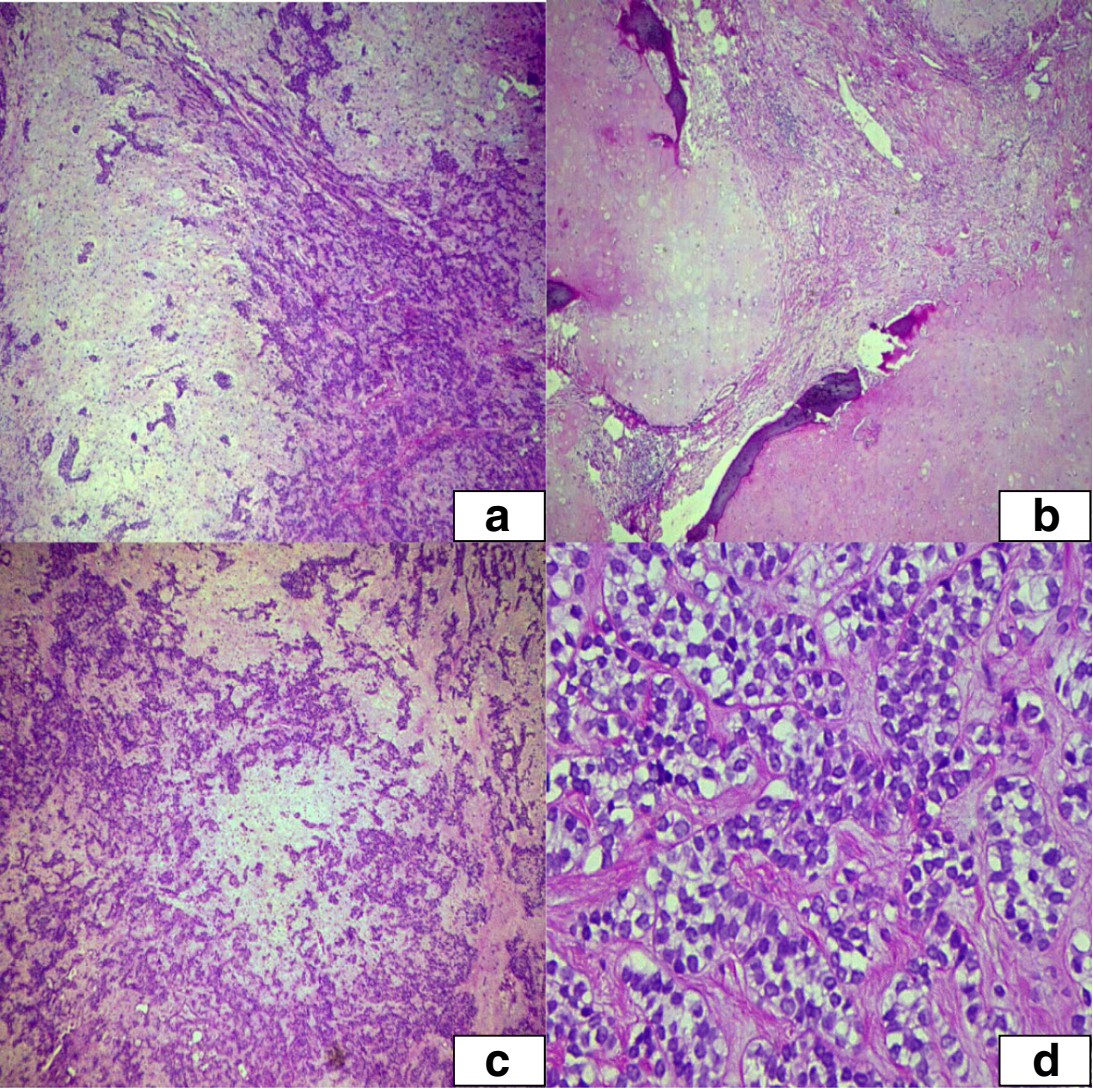
Microscopic sections of tumor **a**. tumor in nests, cords and trabeculae with myxoid background, **b**. Cartilage formation with calcification and ossification, **c** and **d**. higher power showing tumor cells showing no significant atypia or mitosis

**Fig. 3:**
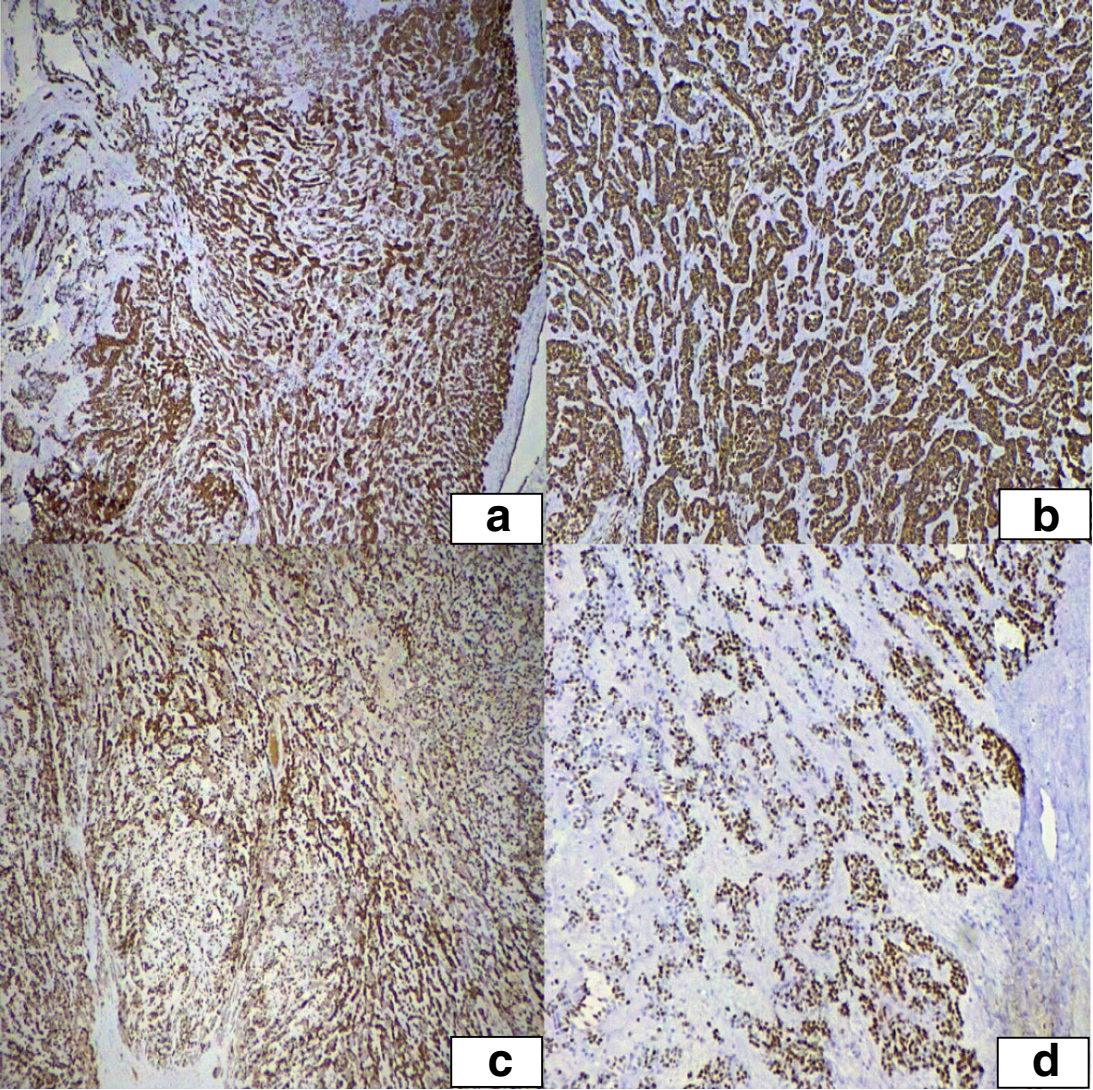
Positive expression with, **a**. CKAE1/AE3 immunostain, **b**. Vimentin, **c**. S100, **d**. p63 immunostains

The specimen received in histopathology department is composed of multiple white glistening nodules of tumor with myxoidy cut surface measuring 20 X 10 cm in aggregate. Twenty two sections from the tumor were submitted. Microscopic examination show neoplastic lesion composed of nests, cords and trabeculae of small to medium sized cells with round nuclei and clear cytoplasm. Background showed myxoid appearance with areas of cartilaginous differentiation. Foci of metaplastic bone formation were also noted. Borders of the tumor were well defined and no invasive into adjacent soft tissue noted. There was not ductal differentiation in tumor cells. No germ cell component (including teratomatous component) noted. No significant atypia, necrosis or mitotic activity was seen in tumor cells (Fig. 2). Immunohistochemical stains were also performed. Tumors cells showed positive expression with CKAE1/AE3, CK7, Vimentin, MIC2, S100, ASMA, p63 and GFAP immunostains while CK20, LCA, Chromogranin A, calretinin, TTF1 and CD5 were negative (Fig. 3). Focal expression of CD117 and WT1 (cytoplasmic) was also noted. On the basis of these morphologic features and immunohistochemical profile a diagnosis of benign myoepithelioma was favored. Cytogenetic studies were not performed. Postoperative course was unremarkable. No recurrence or metastasis was observed at 6 months follow up.

## Discussion

Myepitheliomas and benign mixed tumors are described in thoracic location including lung with confusing terminology [6, 7]. Some authors illustrated cases of pleomorphic adenoma in lung and mediastinum with an assumption that they arise from submucosal glands of respiratory tree or ectopic salivary gland tissue [8, 9]. However occurrence of these tumors unrelated to trachea and bronchi speaks against its origin from submucosal glands, as now it is well known that tumors of this kind can occur anywhere in the body and no site is exempted. Cases described as unusual adnexal tumors in subcutaneous tissue and pleomorphic adenoma in lungs and mediastinum may actually represent myoepitheliomas. In our case, no connection with respiratory mucosa or submucosa was seen and therefore can be assumed that these tumors like in other soft tissue location arise from stem cells capable of divergent differentiation. Histopathologically, these tumors show diverse growth patterns in the form of cords, nests and trabeculae with heterologous stromal components. Immunohistochemical requirement for the diagnosis of myoepithelial tumors is the expression of S100, GFAP or ASMA in addition to cytokeratin stains [10]. The criteria for malignancy in myoepithelial tumors is invasion into adjacent tissues, necrosis, marked nuclear atypia and mitotic activity [11]. None of these features was seen in our case. On the other hand, our case is unique in the aspect that it is quite large in size effacing the structures of thoracic cavity and lungs.

Histopathologic recognition of myoepithelial tumors is very essential as incorrect diagnosis may lead to grave consequences. As in our case, two previous biopsies revealed the diagnosis of chondroid hamartoma and sarcomatoid mesothelioma respectively. The diagnosis of chondroid hamartoma was rendered due to presence of large amount of cartilage mixed with epithelial elements. On the other hand, there was focal expression of WT 1 which led to the misdiagnosis of mesothelioma.

## Conclusion

Myoepithelial tumors are rare soft tissue tumors thought to arise from stem cells capable of divergent differentiation. Histopathologic recognition of these tumors is essential as these tumors may behave in a benign fashion despite large sizes.

## Consent

Written informed consent was obtained from the patient for publication of this Case Report and accompanying images. A copy of the written consent is available for review by the Editor-in-Chief of this journal. Approval obtained from Liaquat national hospital and medical college ethical committee.
